# Klotho Suppresses Indoxyl Sulfate-Mediated Apoptosis in Human Kidney Proximal Tubular (HK-2) Cells through Modulating the AKT/Nrf2 Mechanism

**DOI:** 10.1021/acsomega.4c08038

**Published:** 2025-06-03

**Authors:** Chien-Yao Sun, Yao-Tsung Lin, Yu-Ting Huang, Hui-Ching Cheng, Wan-Ching Chou, Yu-Tzu Chang, Tsai-Chieh Ling, Pei-Ling Hsieh, Kun-Ling Tsai

**Affiliations:** † Department of Geriatric and Gerontology, National Cheng Kung University Hospital, College of Medicine, 34912 National Cheng Kung University, Tainan 704, Taiwan; ‡ Department of Geriatric and Gerontology, College of Medicine, National Cheng Kung University, Tainan 704, Taiwan; § Institute of Allied Health Sciences, College of Medicine, National Cheng Kung University, Tainan 704, Taiwan; ∥ Department of Anesthesiology, 38018 Chi Mei Medical Center, Tainan 710, Taiwan; ⊥ Department of Hospital and Health Care Administration, Chia Nan University of Pharmacy and Science, Tainan 717, Taiwan; # Department of Physical Therapy, College of Medicine, National Cheng Kung University, Tainan 704, Taiwan; ¶ Department of Internal Medicine, National Cheng Kung University Hospital, College of Medicine, National Cheng Kung University, Tainan 704, Taiwan; ∇ Department of Anatomy, School of Medicine, China Medical University, Taichung 404, Taiwan; □ Department of Medicine, University of California San Francisco, San Francisco, California 94143, United States

## Abstract

Chronic kidney disease (CKD) is a progressive condition with substantial prevalence worldwide. The uremic toxin indoxyl sulfate (IS) is known to induce tubulotoxicity and adverse effects in various organs. It has been shown that the expression of the antiaging klotho protein is downregulated in the IS-stimulated proximal tubule cells and kidneys, but the detailed mechanism underlying the implication of reduced klotho in nephropathy remains largely unclear. In the present study, we demonstrated that the repressed klotho following IS stimulation contributed to the reduced cell viability and increased cytotoxicity of HK-2 cells (a proximal tubular cell line). We showed that recombinant klotho reversed the AKT/Nrf2 axis in the IS-treated HK-2 cells, leading to the restoration of the antioxidant HO-1, NQO1, and SOD as well as diminished ROS production. Most importantly, our results suggested that the IS-induced alteration of mitochondrial membrane potential, mitochondrial COX-III mRNA expression, and mitochondrial complex III activity was all mediated by the klotho/AKT/Nrf2 axis. By examining the expression of Bax, Bcl-2, and cytochrome *c* using Western blot and caspase-3-positive cells using flow cytometry, we demonstrated that klotho participated in the IS-induced apoptosis of HK-2 cells as well. Taken together, our data revealed that downregulation of klotho in the IS-stimulated HK-2 cells leads to decreased cell viability as a result of lower antioxidant capacity and ROS accumulation following mitochondrial respiratory chain dysfunction and subsequent apoptosis, possibly through inhibition of the AKT/Nrf2 axis.

## Graphical abstract

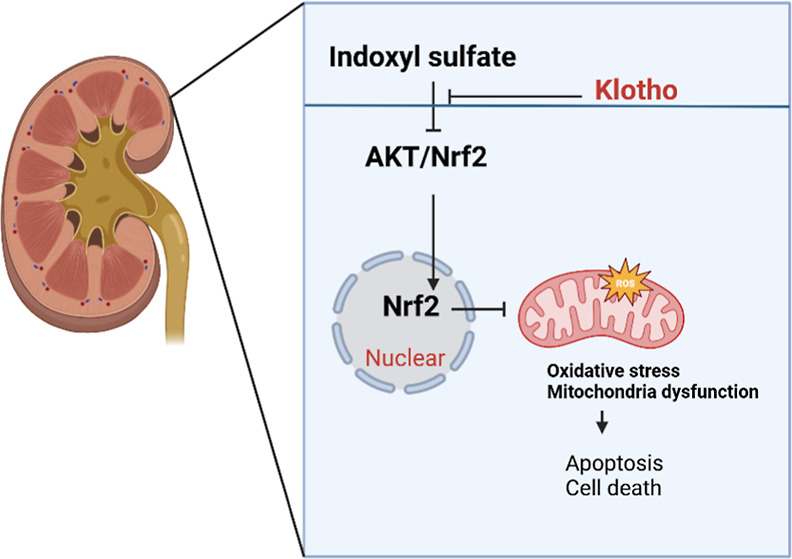

## 1 Introduction

Chronic kidney disease (CKD) has long been considered a global public health problem,^1^ and it has been estimated that the number of patients affected by CKD was more than 800 million worldwide in 2017.^2^ CKD is not only a risk factor for the development of cardiovascular diseases^3^ but also a leading cause of mortality worldwide according to the Global Burden of Disease (GBD) study.^4^ Growing evidence has indicated the significance of the protein-bound uremic retention solutes, such as indoxyl sulfate (IS), in renal disease progression (see review^5^). IS is made by the liver from indole, which was produced from dietary tryptophan by gut-residing bacteria. IS can be taken up into the tubular cells by organic anion transporters (OATs)^6^ and then excreted from the kidney.^7^ Nevertheless, it may remain unfiltered and accumulate in the circulation due to dysfunction of urinary excretion,^8^ resulting in deleterious effects on kidneys and other organs. In addition, sufficient removal of IS is difficult to achieve by hemodialysis due to its strong binding affinity to serum albumin.^9^ It has been demonstrated that IS-induced epithelial-to-mesenchymal transition (EMT) and apoptosis of renal tubular cells contribute to the deterioration of CKD.^10^ Besides, oxidative stress in proximal tubular cells has been shown to induce significantly the activation of nuclear factor-kappa B (NF-κB) after exposure to IS,^11^ leading to cellular senescence.^12^ Although the tubulotoxicity of IS has been well-recognized, the detailed mechanisms underlying the pathophysiology of IS-induced renal damage have not been fully revealed.

The klotho gene has been identified in various tissues, such as the renal proximal^13^ or distal^14^ convoluted tubules, and was originally considered as an aging-suppressor gene.^14^ Klotho family members include α-Klotho, β-Klotho, and γ-Klotho, all of which are single-transmembrane proteins with varying lengths.^15^ They have been known to increase the affinity of the endocrine fibroblast growth factor (FGF) (e.g., FGF19, FGF21, and FGF23) to their cognate FGF receptors.^16^ Aside from serving as a coreceptor of FGFs, klotho proteins have also been discovered to suppress IGF/insulin signaling and Wnt signal transduction and enhance resistance to oxidative stress.^17^

Klotho possesses potential protective activity within the kidney, which can likely be attributed to modulation of the AKT/Nrf2 pathway. Klotho supplementation mitigates renal hypertrophy by modulating the AKT signaling pathway in diabetic models,^18^ alleviates medullary fibrosis, enhances pressure natriuresis in hypertensive rats,^19^ and hinders the progression of renal cell carcinoma.^20^ The protective mechanisms extend to cardiovascular aging, with klotho activating the Nrf2-mediated antioxidant defense pathway in both aged mice^21^ and human aortic smooth muscle cells.^22^ Recombinant human klotho significantly counteracts oxidative stress in retinal pigment epithelial cells by activating the PI3K/Akt-Nrf2/HO-1 signaling pathway.^23^ In the kidneys, klotho may exert modulation through the AKT/Nrf2 pathway, although the evidence remains elusive. In the present study, we hypothesize that klotho could suppress IS-causing kidney proximal tubular (HK-2) cell apoptosis and cellular damage. Our research aimed to examine whether the IS-induced cytotoxicity of HK-2 cells was mediated by the reduction of klotho. In addition, we assessed if downregulation of klotho led to inhibition of AKT/nuclear factor erythroid 2-related factor 2 (Nrf2) signaling followed by downregulation of heme oxygenase-1 (HO-1) and NAD­(P)­H-quinone oxidoreductase 1 (NQO1) as well as an increase in oxidative stress. Furthermore, we investigated whether the klotho/AKT/Nrf2 axis affected apoptosis and mitochondrial dysfunction with regard to membrane potential and members of the mitochondrial respiratory chain in the IS-stimulated cells. Our results shed light on the molecular mechanisms of the IS-mediated exacerbation of renal injury.

## 2 Methods

### 2.1 Cell Culture and Reagents

The renal proximal tubule epithelial cells, HK-2 cells, were obtained from ATCC (PCS-400-010). The base medium for this cell line was a keratinocyte serum-free medium complemented with 0.05 mg/mL bovine pituitary extract (BPE) and 5 ng/mL human recombinant epidermal growth factor (EGF). Cells were cultured in a 37 °C incubator with 5% CO_2_. After 80% confluence, cells were subcultured by 0.05% Trypsin–EDTA. The subcultivation ratio was 1:4. Fetal bovine serum (FBS) and EDTA were obtained from Gibco (NY, USA). 2′,7′-Dichlorofluorescein diacetate (DCFH-DA), 5,58,6,68-tetraethylbenzimidazolcarbocyanine iodide (JC-1), LY294002, ML 385, SC79, *N*-acetyl-l-cysteine (NAC), and indoxyl sulfate (IS) were bought from Sigma-Aldrich (St. Louis, MO, USA). The human recombinant epidermal growth factor (EGF), Klotho, anti-Klotho antibody, and secondary antibodies with HRP conjugated were purchased from Thermo Fisher Scientific (Waltham, MA, USA). Anti-Bcl-2, anti-Bax, anti-Cytochrome C, anti-PCNA, anti-β-actin, antiphospho-AKT, anti-AKT, and anti-Nrf2 antibodies were obtained from Cell Signaling Technology (Danvers, MA, USA).

### 2.2 Cell Viability and Cytotoxicity

Cell viability was evaluated using an MTT assay. After stimulation of IS (500 μM) for 24 h, the cells were cultured in Keratinocyte Serum Free Medium and treated with a concentration of 0.5 mg/mL MTT for a duration of 1 h. Following incubation, the medium was aspirated and the blue MTT-formazan product was solubilized using dimethyl sulfoxide (DMSO). The absorbance of the resulting formazan solution was measured by using a spectrophotometer at a wavelength of 570 nm. According to the assay protocol, the LDH Assay Kit (Abcam; ab65393) tested the cytotoxicity. In brief, after stimulation of IS, cell-free culture medium (10 μL) and the LDH Reaction Mix (100 μL) were loaded into each well for 30 min of incubation. The absorbance was 450 nm, and the reference wavelength was 650 nm.

### 2.3 Western Blotting Assay

Cells were lysed in ice-cold 1× RIPA. The samples were centrifuged at 12,000 rpm for 15 min at 4 °C. We used 10% SDS-polyacrylamide gel for electrophoresis and then transferred the proteins to PVDF membranes. After blocking the blots with 5% milk (in PBST), the membranes were washed with PBST and incubated with primary antibodies overnight at 4 °C. Then, we washed those membranes with PBST twice and incubated the HRP-conjugated secondary antibodies. ECL was used as the chemiluminescent Western blotting substrate. The proteins were quantified using ImageJ software.

### 2.4 RNA Isolation and Real-Time PCR

Following cell collection, chloroform was added to the TRIzol-sample mixture, which was then centrifuged to separate the mixture into aqueous and organic phases. The RNA was recovered from the aqueous phase. Isopropanol was added to the recovered RNA-containing aqueous phase and centrifuged to precipitate RNA. The resulting RNA pellet was washed with 75% ethanol and air-dried before being resuspended in RNase-free water. Additionally, the RNase inhibitor was added to prevent RNA degradation. Real-time PCR was performed using the ABI StepOnePlus Real-Time PCR System. Each 20 μL PCR reaction included 10 μL of 2X SYBR Green PCR Master Mix (ABI, USA), 1 μL of cDNA, and 0.5 μM of each primer. The following primer pairs were used for PCR amplification: Klotho: F, 5′-ACTCCCCCAGTCAGGTGGCGGTA-3′; R, 5′-TGGGCCCGGGAAACCATTGCTGTC-3′; β-actin; F:5′-CCCTGGCTCCTAGCACCAT-3′; R:5′-GATAGAGCCACCAATCCAATCCACACA-3′. COX III; F: 5′-CTAAACACATCCGTATTACTCGCAT-3′ ; R: 5′-TCGGAAATGGTGAAGGGAGAT-3′. The amplification conditions were 95 °C for 10 min, followed by 40 cycles of 95 °C for 15 s and 60 °C for 1 min. A final melt curve analysis was conducted from 65 to 95 °C to confirm the specificity of the amplification. The relative expression levels of β-actin were calculated using the 2^(−ΔΔCt)^ method, with β-actin serving as the internal control.

### 2.52.5 ROS Concentration

Polystyrene 96-well microplates were seeded with 2.5 × 10^4^ HK-2 cells for 24 h of incubation with a completed medium before IS stimulation. At the end of IS stimulation, cells were washed with 1 × PBS twice and stained with 5 μM of 2′,7′-dichlorofluorescin diacetate (DCF-DA) (in culture medium) for 30 min at 37 °C. The ROS levels were measured using a fluorescence microplate reader (*E*_x_/*E*_m_ = 485/535) after washing with 1 × PBS.

### 2.6 Nrf2 Activity Assay, Mitochondrial Complex III Activity Assay, and SOD Activity

The activity of Nrf2 was examined by an NRF2 Transcription Factor Activity Assay Kit (BioVision; E4337-100). The nuclear extraction was loaded to each well and incubated for 2 h at 37 °C with gentle shaking. Then, the solution was removed and washed 4 times with wash buffer. Nrf2 primary antibodies (100 μL) were loaded into each well for 1 h, repeating the washing step before HRP-conjugated secondary antibody incubation. The final step was loading the stop solution before incubation with the 3, 3′, 5, 5′-tetramethylbenzidine (TMB) one-step substrate reagent for 30 min. The O.D. was 450 nm for the detection of the Nrf2 activity. The Mitochondrial Complex III activity was studied with the Mitochondrial Complex III Activity Assay Kit (Abcam; ab287844). We isolated the mitochondria from cells with a mitochondria isolation kit. The reduced cytochrome c standard curve was calculated with the mitochondrial complex III activity assay. The SOD activity was tested by the CuZn/Mn Superoxide Dismutase (CuZn-SOD/Mn-SOD) Activity Assay Kit (Elabscience; E-BC-K022) according to the manufacturer’s protocol.

### 2.7 Assessment of the Mitochondrial Membrane Potential

The mitochondrial membrane potential was evaluated using the 5,5′,6,6′-tetrachloro-1,1′,3,3′-tetraethylbenzimidazolylcarbocyanine iodide (JC-1) fluorescence dye. Cells were stained with a JC-1 working solution and incubated at 37 °C for 30 min in a light-protected environment. Subsequently, cells were washed with PBS, and the fluorescence signal was measured by using a microscope. For flow cytometry analysis, cells were harvested, washed with PBS, and resuspended in a medium containing JC-1. After incubation at 37 °C with 5% CO_2_, the cells were subjected to flow cytometry analysis using a FACSAria III flow cytometer (Becton Dickinson, Heidelberg, Germany).

### 2.8 Transfection with Small-Interfering RNA

ON-TARGET plus SMART pool small-interfering RNAs (siRNAs) for si-Control were obtained from Dharmacon Research (Lafayette, CO, USA). si-Nrf2 was purchased from Santa Cruz. According to the manufacturer’s guide, transfection was performed using an INTERFERin siRNA transfection reagent (jetPRIME, polyplus transfection, UK). The efficiency of siRNA knockdown was confirmed by Western blotting. The protein expression of klotho reveals almost 70% reduction after 48 h of si-Nrf2 transfection (data not shown).

### 2.9 Apoptosis Assay

The Caspase-3 (active) Red Staining Kit was used for the caspase 3 assay (Abcam; ab65617). Cells were collected in Eppendorf tubes. 300 μL of 1× wash buffer and 1 μL of Red-DEVD-FMK were loaded into each tube and incubated for 1 h in a 37 °C incubator with 5% CO_2_. After incubation, cells were washed twice with a 1× wash. The cells underwent flow cytometry analysis by using a FACSAria III flow cytometer. The BD Pharmingen FITC Annexin V Apoptosis Detection Kit was used for identifying apoptotic cells.

### 2.10 Cytosol Isolation

Cytosol protein isolation was conducted by the Mitochondria/Cytosol Fractionation Kit (Abcam, ab65320). After stimulation of IS (500 μM) for 24 h, cells were collected using trypsin and centrifugation at 1200 rpm for 5 min at 4 °C. After washing with cold PBS, 1 mL of Cytosol Extraction Buffer containing proteinase inhibitor and DTT was resuspended with pellets. An ice-cold Dounce tissue grinder was used to homogenize the cells. After 10 min of incubation and 50 passes, samples were transferred to a 1.5 mL microcentrifuge tube and centrifuged at 3000 rpm for 15 min at 4 °C for supernatant collection (cytosol protein).

### 2.11 Statistical Analysis

The data are presented as mean ± standard deviation (SD) from a minimum of three independent experiments per group. Statistical analysis was performed using one-way analysis of variance (ANOVA) followed by the Tukey post hoc test to determine differences among groups. A significance level of *P* < 0.05 was considered statistically significant.

## 3 Results

### 3.1 Exposure to IS Reduces Cell Viability of HK-2 Cells via Suppression of Klotho Protein

We first analyzed the expression of klotho in an immortalized proximal tubule epithelial cell line, HK-2 cells. As shown in Figure 1A, real-time PCR was conducted to study the expression of klotho mRNA under 500 μM IS stimulation; the dosage of IS is referred to in previous publications.^24^ Stimulation of 500 μM also suppressed ∼47% klotho mRNA expression. We also observed that IS stimulation inhibited ∼45% klotho protein levels (Figure 1B,C). In addition, we observed that cell viability was ∼38% inhibited when they were treated with 500 μM IS, whereas administration of 100 or 200 ng/mL klotho reversed it (Figure 1D). We also assessed the release of lactate dehydrogenase (LDH) to evaluate cytotoxicity and showed that it was approximately 3.5 times higher in cells treated with 500 μM IS than in the control group. Similarly, we found that administration of 100 or 200 ng/mL klotho markedly attenuated the release of LDH of HK-2 cells in response to 500 μM IS (Figure 1E). These results indicated that IS decreased cell viability and increased cytotoxicity of HK-2 cells were associated with the downregulation of the klotho protein.

**Figure 1:**
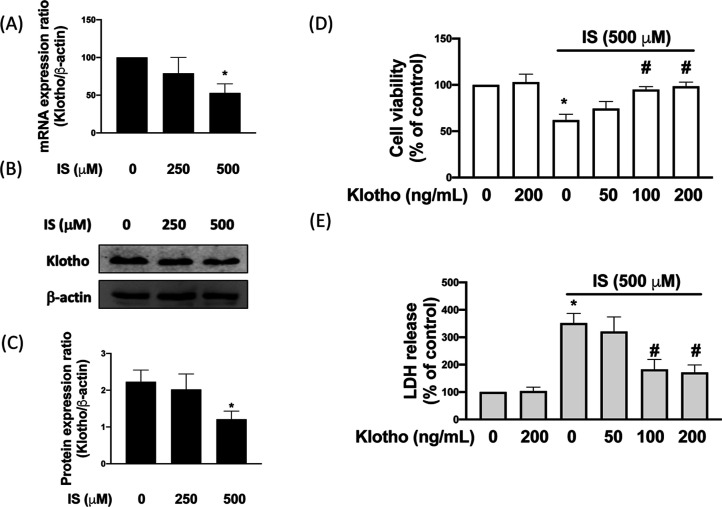
Klotho reverses IS-reduced cell viability in HK-2 cells. The mRNA (A) and protein (B,C) expression levels of Klotho in HK-2 treated with IS were reduced in a dose-dependent manner. Cell viability was investigated using the MTT assay (D). The LDH assay was used to study the IS-mediated cytotoxicity (E). Results were expressed as means ± SD of three independent experiments. (* indicating *p* < 0.05 compared with the control group; # indicating *p* < 0.05 compared to IS-stimulated cells).

### 3.2 IS Induces Downregulation of the AKT/Nrf2 Axis through the Reduction of the Klotho Protein

A couple of studies have demonstrated that the klotho protein protects against hydrogen peroxide-induced oxidative injury in endothelial cells^25^ or retinal pigment epithelial cells^23^ via AKT/Nrf2 axis. Given that the IS-elicited reactive oxygen species (ROS) production is implicated in the progression of CKD^26^ and klotho may exert its protective effect through the AKT/Nrf2 axis, we then examined the expression of AKT and Nrf2 following IS treatment. We showed that the phosphorylation of AKT was ∼73% inhibited following IS stimulation, while recombinant klotho abrogated this effect (Figure 2A and B). As for Nrf2, we found a similar finding that recombinant klotho can restore the IS-inhibited ∼80% Nrf2 expression (Figure 2C and D). Moreover, the administration of the AKT activator, SC79, also abolished the suppressive effect of IS on Nrf2 expression (Figure 2C,and D), suggesting that IS may diminish the Nrf2 expression through inhibition of klotho and AKT. In addition, the result of Nrf2 activity also supported the involvement of klotho and AKT. We demonstrated that treatment of recombinant klotho enhanced Nrf2 activity in the IS-stimulated cells (0.42 VS. 0.88, *p* < 0.05), but this phenomenon was eliminated when the AKT inhibitor, LY2780301, was present (0.88 VS. 0.52, *p* < 0.05) (Figure 2E). Likewise, activation of AKT using SC79 blocked the inhibitory property of IS on the Nrf2 activity (Figure 2E). Accordingly, our results demonstrated that the reduced Nrf2 expression and activity in HK-2 cells following IS stimulation may result from the repression of the klotho/AKT/Nrf2 axis.

**Figure 2:**
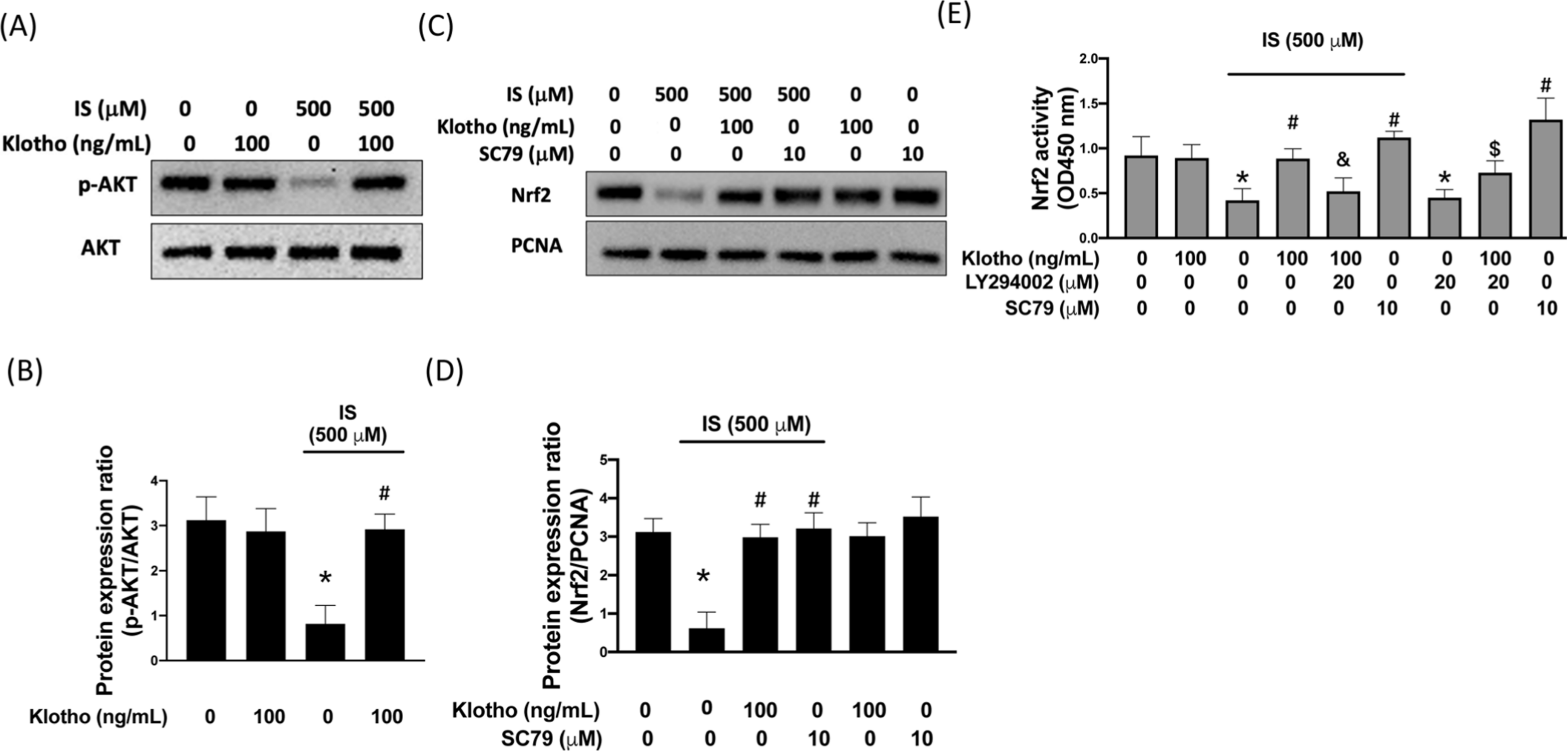
Klotho mitigates IS-suppressed AKT and Nrf-2 expression. Representative Western blot images and relative densitometric bar graphs of p-AKT/AKT (A,B) and Nrf-2/β-actin (C,D) in HK-2 cells stimulated to 500 μM IS for 24 h are shown. Klotho reverses IS-mitigated Nrf-2 activity (E). Results are expressed as means ± SD of three independent experiments. (* indicating *p* < 0.05 compared with the control group; # indicating *p* < 0.05 compared to IS-stimulated cells; & indicating *p* < 0.05 compared to IS-stimulated plus Klotho cells).

### 3.3 IS-Induced Oxidative Stress Is Mediated by the Inhibited Antioxidant Capacity via the Klotho/AKT/Nrf2 Pathway and Elevation of ROS

After translocating to the nucleus and binding to the antioxidant response element (ARE), Nrf2 drives the expression of various target genes, such as HO-1 and NQO1.^27^ Here, we observed that the mRNA expression levels of HO-1 (∼57% reduction) and NQO1 (∼65% reduction) were both suppressed in IS-treated cells, whereas administration of klotho counteracted them (Figure 3A and B). Blockage of AKT/Nrf2 signaling via the AKT inhibitor (LY2780301) or silencing of Nrf2 both impeded the rescued effect of klotho on HO-1 and NQO1 expression, suggesting that IS inhibited the expression of HO-1 and NQO1 via the klotho/AKT/Nrf2 axis. On the other hand, the induction of these antioxidant factors is presumed to confer resistance to oxidative stress by upregulation of various antioxidant enzymes. Hence, we examined the expression of superoxide dismutase (SOD) and found that the concentration of SOD was ∼57% reduced subsequent to IS treatment, which was restored by recombinant klotho (∼16% reduction compared to the control group; Figure 3C). Likewise, inhibition of the AKT/Nrf2 signaling offset the beneficial effect of klotho on the SOD concentration (∼52% and ∼62% reduction compared to the IS + klotho group) (Figure 3C). Unsurprisingly, the ROS formation was 3.2-fold elevated in the IS-stimulated cells, while recombinant klotho hindered the upregulation of ROS. The antioxidant NAC also mitigated IS-increased ROS concentrations (Figure 3D and E). Our results showed that klotho defended against IS-induced ROS production. However, this finding was reversed by inhibiting AKT/Nrf2 signaling. Altogether, these findings suggested that IS-elicited the upregulation of oxidative stress in HK-2 cells through elevation of ROS and inhibition of the antioxidant capacity, as evidenced by lower expression of HO-1, NQO1, and SOD via the klotho/AKT/Nrf2 signaling.

**Figure 3:**
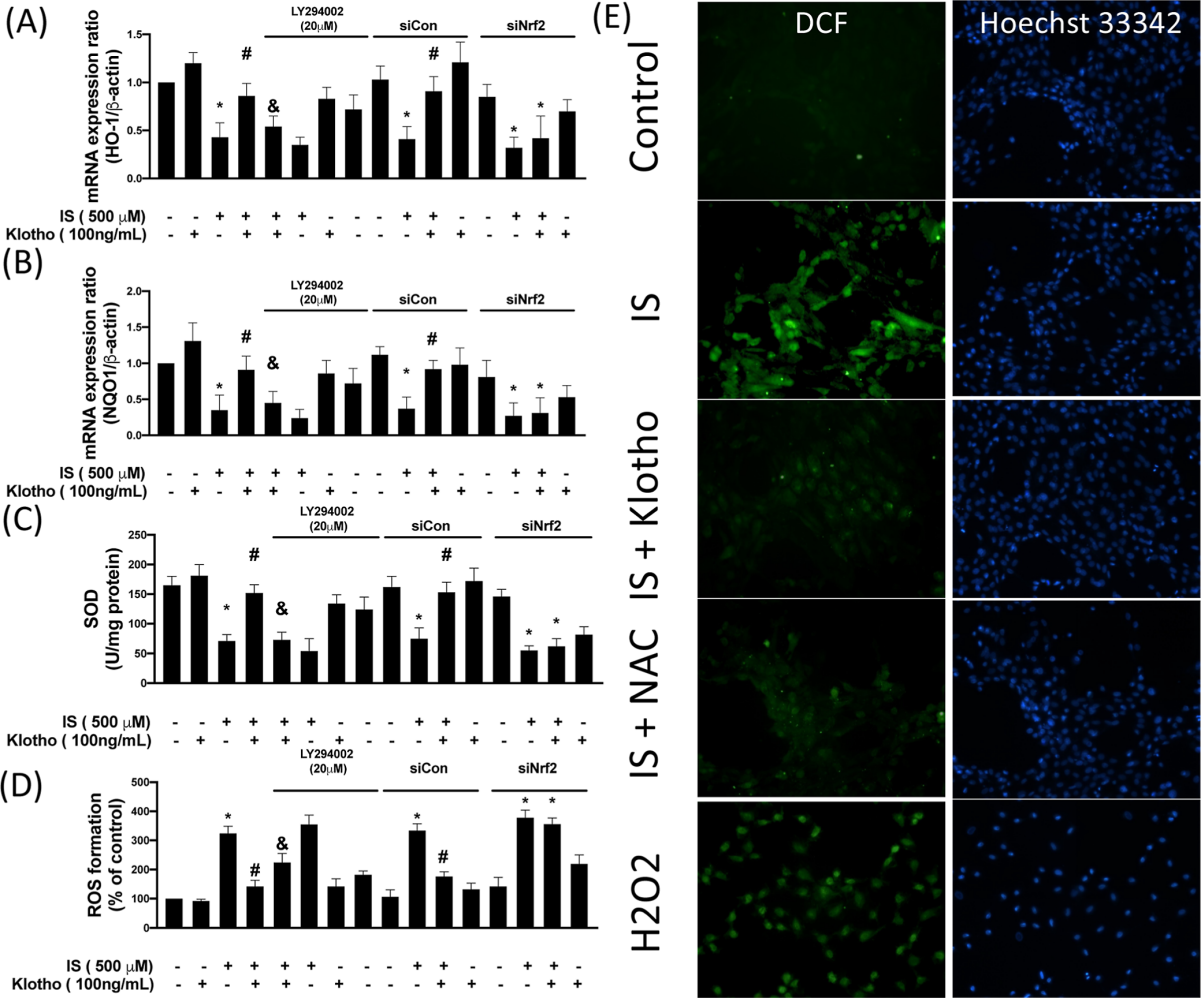
Klotho reduces IS-increased oxidative stress. HK-2 cells were exposed to 500 μM IS for 24 h. In some cases, siControl (siCon) and siNrf2 were transfected for 48 h before IS stimulation. LY264002 was pretreated for 2 h before IS stimulation. HO-1 (A) and NQO-1 (B) mRNA expression levels were investigated by the real-time PCR. An SOD activity assay was used to determine the activity of the antioxidant enzyme (C). ROS concentration was investigated by DCF-DA. The bar chart represents the fluorescence intensity (D). The fluorescence microscopy images were also studied (E), and NAC was used for antioxidant positive control. H2O2 (100 μM) was used for the positive control of ROS. Results are expressed as means ± SD of three independent experiments. (* indicating *p* < 0.05 compared with the control group; # indicating *p* < 0.05 compared to IS-stimulated cells; & indicating *p* < 0.05 compared to IS-stimulated plus Klotho cells).

### 3.4 IS-Elicited Mitochondrial Dysfunction in HK-2 Cells Is Mediated by the Klotho/AKT/Nrf2 Axis

It is well-accepted that ROS production is associated with the dysregulation of the oxidative phosphorylation (OXPHOS) system and mitochondria abnormalities have been discovered in patients with CKD.^28^ One way to discriminate the energized and deenergized mitochondria is to use a cationic carbocyanine dye, JC-1, which forms red fluorescent aggregates when concentrated in healthy mitochondria. Figure 4A and B demonstrated a reduction of healthy cells expressing JC-1 red fluorescence (FL2) after exposure to IS (FL2 45%). With the treatment of klotho, the green fluorescence emission (FL1) was quenched (FL1 6.5%), indicating that the unhealthy or apoptotic cells were downregulated (Figure 4B). Nonetheless, this effect was weakened when the AKT inhibitor (LY2780301) (FL1 45%) or Nrf2 inhibitor (ML385) (FL1 47%) was applied (Figure 4B), suggesting the favorable property of klotho required the involvement of AKT/Nrf2 activation.

**Figure 4:**
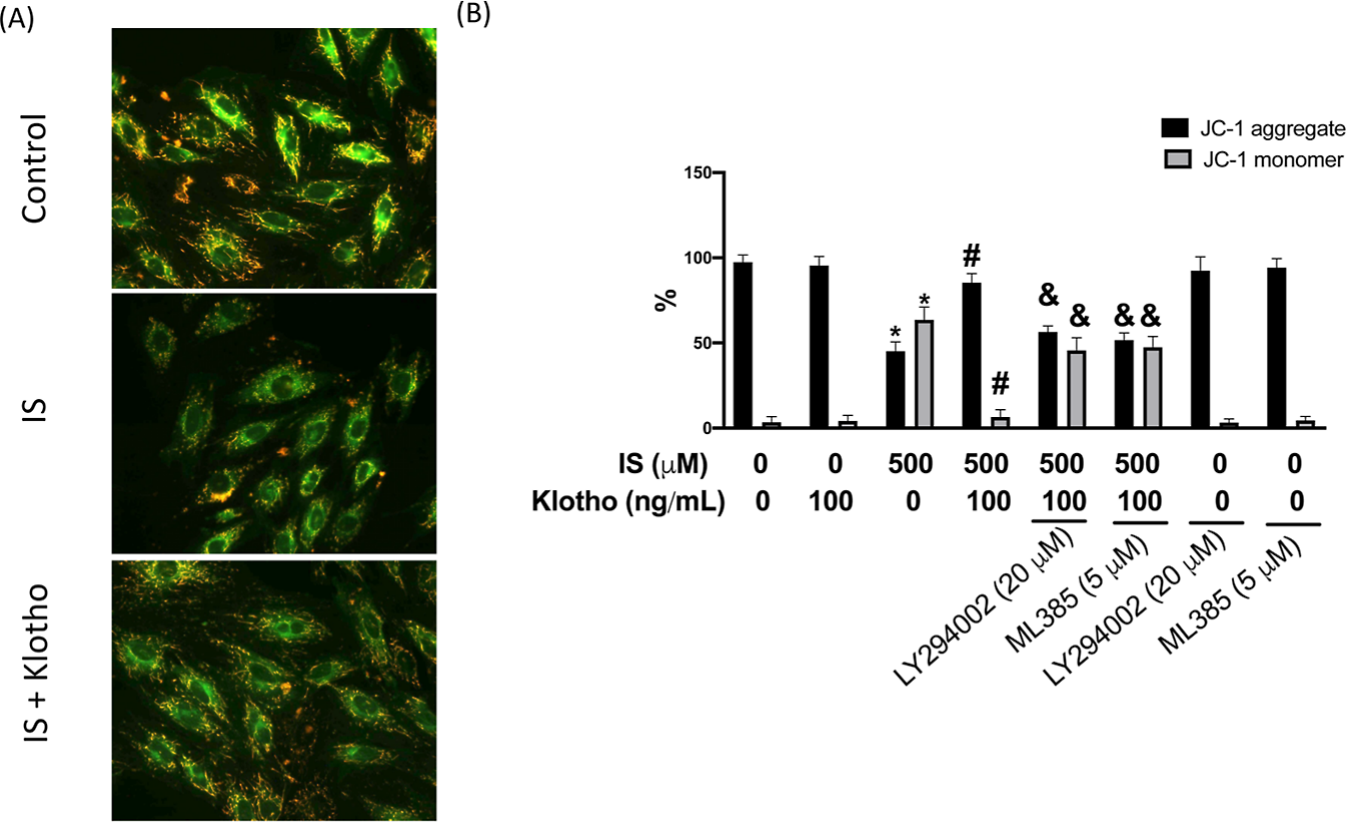
Klotho reverses IS-impaired mitochondrial membrane potential. HK-2 cells were exposed to 500 μM IS for 24 h. In some cases, LY264002 or ML 385 was pretreated for 2 h before IS stimulation. Cells expressing JC-1 aggregates (red fluorescence; FL2) and JC-1 monomers (green fluorescence; FL1) were assessed using a fluorescence microscope (A) and flow cytometry (B). Results are expressed as means ± SD of three independent experiments. (* indicating *p* < 0.05 compared with the control group; # indicating *p* < 0.05 compared to IS-stimulated cells; & indicating *p* < 0.05 compared to IS-stimulated plus Klotho cells).

Cytochrome c oxidase (COX) is the terminal enzyme of the mitochondrial respiratory chain and participates in the transfer of protons, which contributes to the membrane potential. It is built up with both nucleus- and mitochondrion-encoded subunits,^29^ and the mitochondrial origin COXIII is a common biomarker for nephropathy.^30–32^ We showed that the induction of COXIII mRNA expression in the IS-stimulating cells was blocked in the presence of klotho (∼40% inhibition). However, inhibition of AKT/Nrf2 signaling abrogated the effect of klotho on COXIII suppression (∼11% and ∼6% inhibition) (Figure 5A). Additionally, we examined the activity of Complex III as it was at the center of the respiratory chain and vital to catalyze the electron transfer from coenzyme Q to cytochrome c. We found that Complex III activity was ∼65% inhibited in cells treated with IS, whereas klotho mitigated this effect (∼20% inhibition compared to that in the control group). However, the restoration of Complex III activity was not seen in cells cotreated with AKT or Nrf2 inhibitors (∼52% and ∼64% inhibition compared to the control group) (Figure 5B). Collectively, these data demonstrated that IS stimulation may interfere with the electron transport process of Complex III by downregulating the klotho/AKT/Nrf2 axis, resulting in a decline of mitochondrial function.

**Figure 5:**
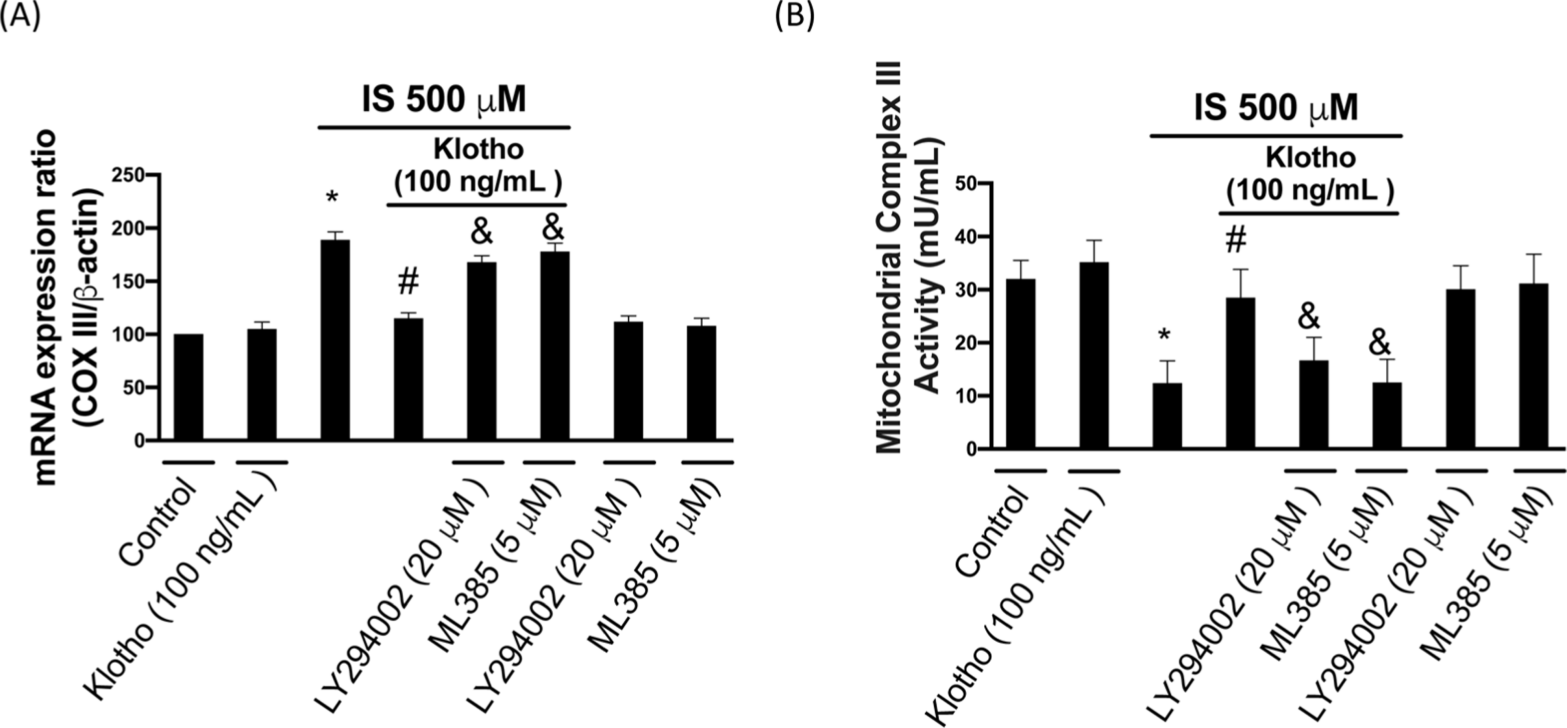
Klotho reverses IS-impaired mitochondrial cytochrome c oxidase III expression. The real-time PCR investigated COX III (A) mRNA expression levels. The COX III activity was studied via the commercial kit (B). Results are expressed as means ± SD of three independent experiments. (* indicating *p* < 0.05 compared with the control group; # indicating *p* < 0.05 compared to IS-stimulated cells; & indicating *p* < 0.05 compared to IS-stimulated plus Klotho cells).

### 3.5 Klotho Protein Protects against the IS-Caused Apoptosis in HK-2 Cells

Given that mitochondrial dysfunction and oxidative stress eventually trigger apoptosis, we analyzed the expression of Bax, Bcl-2, and cytosolic cytochrome c. Results from Western blot showed that IS upregulated the expression of proapoptotic Bax (2.3-fold) and cytochrome c (6-fold) with downregulation of prosurvival Bcl-2 (0.4-fold). With recombinant klotho, these alterations were abolished (Figure 6A–D). From the cell counts plot, we observed the caspase-3-positive cells were increased in the IS-treated cells compared to control cells, while the administration of recombinant klotho attenuated the number of apoptotic cells (Figure 6E). The antiapoptotic effect of klotho was further confirmed using Annexin V assay; the NAC was used as the antioxidant control (Figure 6F). Taken together, these findings demonstrated that IS-caused apoptosis in HK-2 cells may be due to the downregulation of klotho and through modulation of the AKT/Nrf2/oxidative stress mechanism.

**Figure 6:**
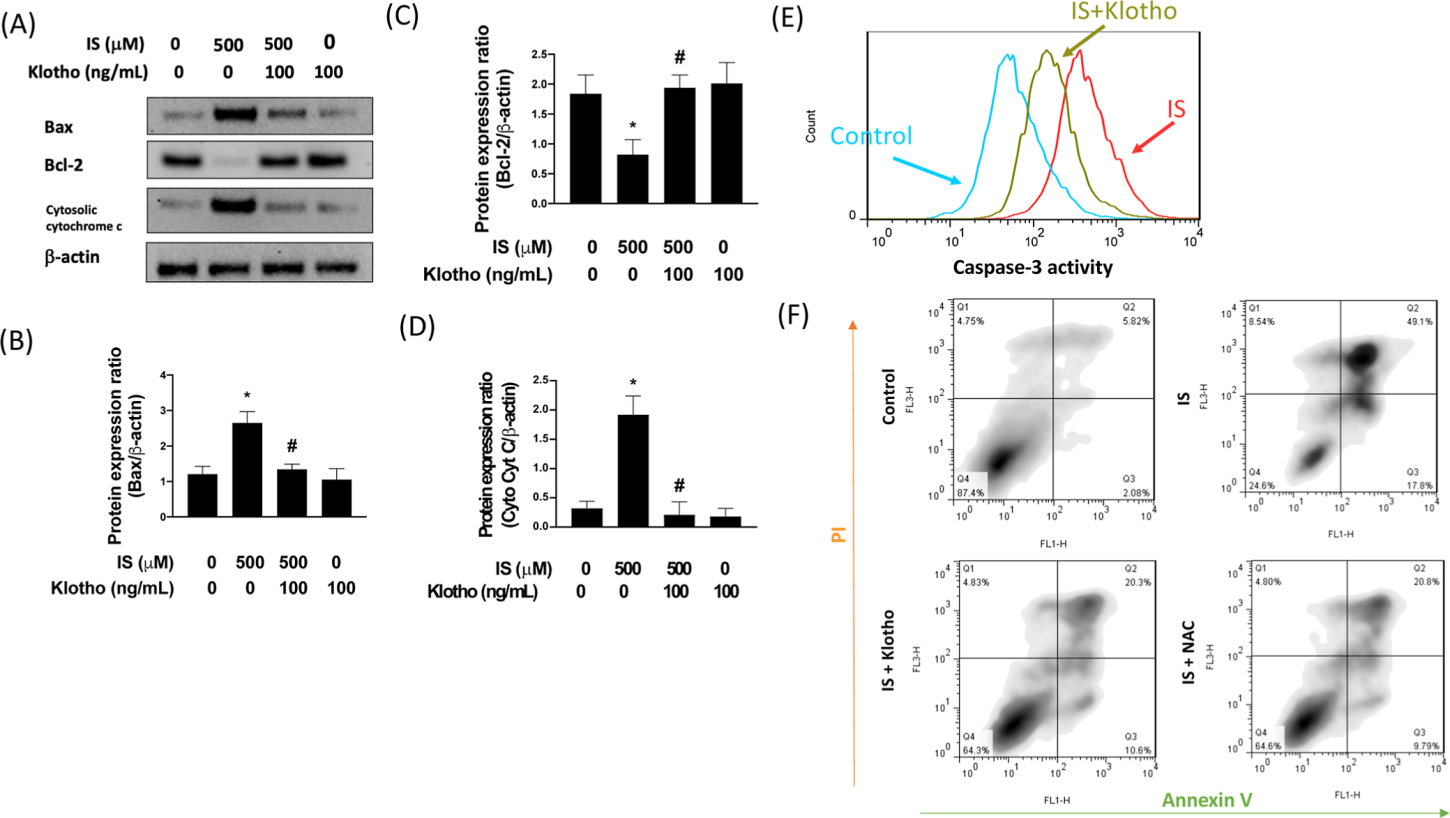
Klotho mitigated IS-induced apoptotic responses. HK-2 cells were exposed to 500 μM IS for 24 h. Representative Western blot images and relative densitometric bar graphs of Bcl-2/b-actin, Bax/b-actin, and cytosolic cytochrome c/b-actin (A–D) in HK-2 cells stimulated to 500 mM IS for 24 h are shown. The activity of caspase 3 was assessed using flow cytometry (E). Annexin V was conducted for apoptosis assay (F). Results are expressed as means ± SD of three independent experiments. (* indicating *p* < 0.05 compared with the control group; # indicating *p* < 0.05 compared to IS-stimulated cells).

## 4 Discussion

IS is a well-known uremic toxin that is elevated in patients with CKD as a result of poor urinary clearance.^33^ Insufficient excretion of IS due to renal dysfunction may further aggravate the IS-induced nephrotoxicity as an association between serum IS and renal progression in CKD patients was reported.^34^ It has been revealed that the upregulation of serum IS may cause IS retention within the proximal tubules via OAT1 and OAT3, and administration of IS resulted in cell death of OAT1- and OAT3-expressing proximal tubular cells.^6^ Also, IS has been found to elicit EMT and apoptosis of renal tubular cells via activation of ERK1/2, p38MAPK,^10^ mTORC1,^35^ or PI3K/Akt^36^ signaling. Another study demonstrated that IS induced the expression of the EMT-associated transcription factor Snail in proximal tubular cells along with the activated TGF-β pathway.^37^ Accumulation of IS may also alter immune response since IS has been demonstrated to stimulate macrophage function and enhance inflammatory response.^35,38^ Besides, IS has also been shown to elicit the production of intracellular and extracellular ROS in cultured mesangial cells^39^ and reduce superoxide scavenging activity in the kidneys of normal rats.^40^ Excessive buildup of ROS in IS-treated proximal tubular cells has been shown to activate NF-κB,^11^ leading to cellular senescence and reduced proliferation.^12^ In line with these studies, we found that exposure of HK-2 cells to IS downregulated cell viability and antioxidant capacity and elevated ROS accumulation due to the reduction of the klotho protein (Figure 7).

**Figure 7:**
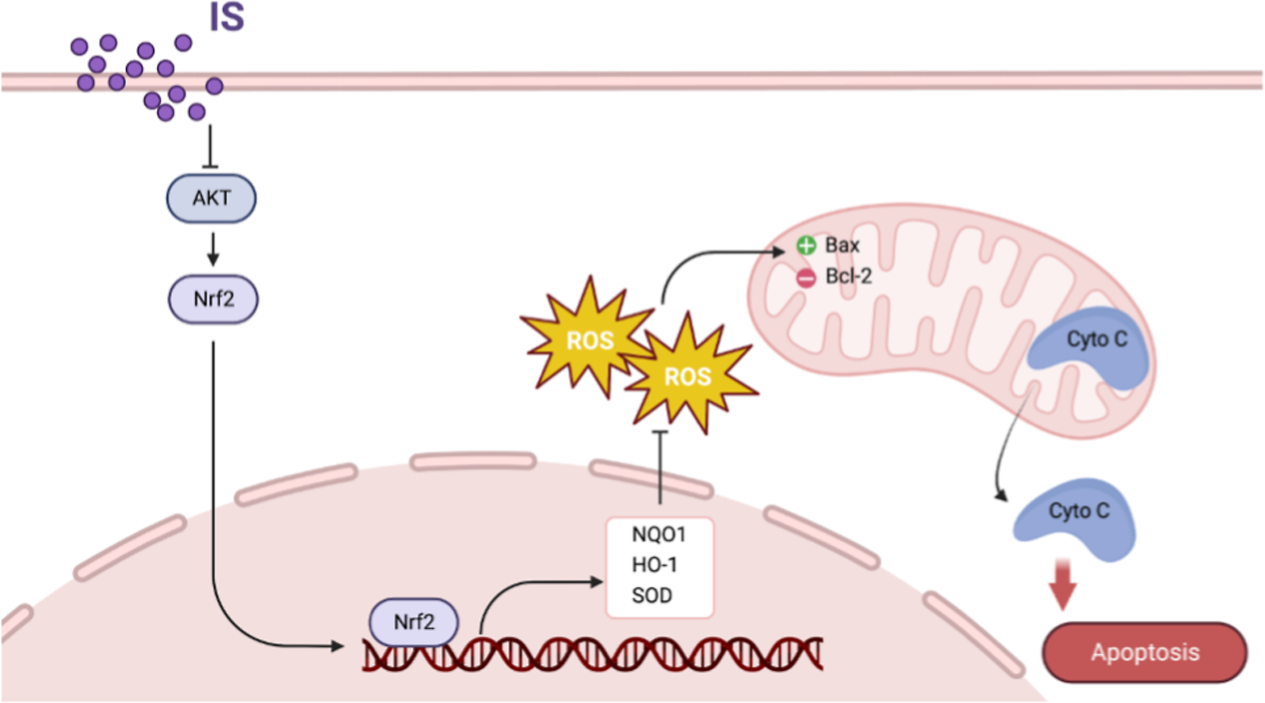
Schematic illustration of this study. Schematic representation of the mechanism by which AK/Nrf2 may mediate the protective role of klotho in indoxyl sulfate-treated proximal tubular HK2 cells against oxidative stress and mitochondrial dysfunction.

Numerous studies have revealed the reduction of klotho in patients with chronic renal failure, IS-stimulated HK-2 proximal tubule cells, and rat kidneys. A klotho deficiency has been found to serve as an early biomarker of renal ischemia–reperfusion injury.^41^ It has been shown that administration of AST-120, an adsorbent of uremic toxins, restored the klotho expression and inhibited cell senescence in the kidney of chronic renal failure rats.^42^ Lack of klotho in proximal renal tubules affected the ability of renal phosphate excretion,^43^ causing vascular calcification^44^ and renal interstitial fibrosis^45^ in vivo. Additionally, the decline in klotho was associated with hyperglycemia and may increase inflammation in the kidney of the db/db mouse model of diabetes.^46^ Emerging studies have uncovered the mechanisms underlying the decline of klotho-Ss following IS stimulation. For instance, Shimizu et al. showed that IS attenuated the expression of klotho through the production of ROS and activation of NF-κB in proximal tubular cells.^47^ IS also increased DNA methylation of the klotho gene and decreased klotho protein expression in HK-2 cells treated with IS and in renal tubules of mice injected with IS.^48^ Our results further demonstrated that the downregulation of klotho was not only a consequence of IS-induced ROS but also a contributor to hastening ROS accumulation.

Emerging evidence indicates that IS downregulates klotho expression in the human kidney-2 (HK-2; proximal tubule epithelial) cells, rat kidneys,^47^ and patients with chronic kidney disease,^49^ exacerbating cellular aging and fibrosis.^50^ This reduction in klotho, a critical antioxidant protector, intensifies renal dysfunction by promoting inflammatory responses, NF-κB activation,^47^ and macrophage M2 polarization.^51^ Considering klotho’s role in antioxidant capacity and amelioration of IS-induced endothelial cell dysfunction,^52^ myocardial hypertrophy,^53^ and kidney damage,^51^ we sought to investigate how the decreased klotho expression underlies the IS-elicited oxidative stress and tubulotoxicity. In addition, recombinant Klotho protein has been reported to be both safe and beneficial, with the therapeutic effects of Klotho lasting up to 12 weeks after intervention in CKD animals.^54^

The significance of klotho has been verified in various IS-associated damage, such as endothelial cell dysfunction,^52^ cardiomyopathy,^53,55^ systemic inflammation,^56^ and kidney damage.^51^ The human physiological concentration of plasma is 71,050 pg/mL–32589.4 pg/mL. In addition, plasma circulating klotho concentration could predict long-term macrovascular outcomes in clinical DM cases.^57^ Besides, it has been shown that the presence of klotho ameliorates the IS-induced endothelial dysfunction via ROS/p38MAPK and NF-κB pathways,^52^ cardiomyocyte hypertrophy via ROS/p38MAPK and ERK1/2 pathways,^53^ and systemic inflammation via RIG-1/NF-κB activation and monocyte inflammatory factor release.^56^ Besides, soluble klotho can protect against cardiac hypertrophy by suppressing abnormal calcium signaling, instead of being a coreceptor for FGF23.^55^ Klotho has also been revealed to possess the ability to counteract IS-elicited platelet hyperactivity by suppressing the ROS/p38MAPK pathway, and Yang et al. showed that klotho protein treatment can attenuate the IS-induced thrombosis and atherosclerosis in apoE–/– mice.^58^ In addition, overexpression of klotho alleviated IS-induced kidney fibrosis and cardiac hypertrophy by inactivating NF-kB signaling and diminishing inflammatory response.^51^ Apart from the ROS/p38MAPK, NF-κB, or ERK1/2 pathways, our data suggested that the reduction of klotho by IS stimulation may engender tubulotoxicity via the AKT/Nrf2 axis.

AKT is a downstream effector of klotho in multiple studies. For example, exogenous klotho supplementation may mitigate renal hypertrophy in db/db mice by downregulating AKT/mTOR signaling.^18^ Klotho administration can also restore the autoregulatory ability of glomerular filtration rate and suppress medullary fibrosis, possibly through the AKT/mTOR pathway in spontaneously hypertensive rats.^19^ Besides, klotho exhibited its antitumor capacity via PI3K/AKT signaling in renal cancer.^20^ Reduced Klotho expression aggravated the tacrolimus-induced renal injury via the PI3K/AKT/FoxO pathway,^59^ and promotion of klotho by ginseng treatment contributed to the enhancement of the binding of FoxO3a to the MnSOD promoter and an increase in MnSOD expression in the mitochondria and mitochondrial function.^60^ Aside from affecting FoxO3a, our results showed that upregulation of klotho activated Nrf2 signaling via AKT, which subsequently triggered the antioxidant defense system. Several studies have shown that klotho alleviates the hydrogen peroxide-induced oxidative injury in endothelial cells^25^ and retinal pigment epithelial cells^23^ via the AKT/Nrf2 axis. Loss of klotho elicited excessive cardiac oxidative stress, senescence, and apoptosis in old mice and klotho mutant mice via the Nrf2/glutathione reductase axis,^21^ and Nrf2 deficiency further exacerbated renal calcification in klotho mutant mice.^61^ The klotho/Nrf2-mediated antioxidant defense also compensated for the damage in the angiotensin II-stimulated vascular smooth muscle cell dysfunction,^22^ high glucose-cultured podocytes, and diabetic db/db mice.^62^ This study reveals new insights into the intricate interplay between klotho and the AKT/Nrf2 pathway under IS-induced oxidative stress. Our findings indicate that IS attenuates the AKT/Nrf2 axis by klotho mitigation, leading to a decreased expression and activity of Nrf2 and reduced antioxidant capabilities. This mechanism is critical in understanding how reduced klotho levels exacerbate oxidative stress and lead to cell death.

Most cellular ROS is predominantly generated by mitochondria, contributing to the development and progression of CKD.^63^ Emerging evidence has suggested that the deficiency of klotho may render the kidney susceptible to damage following mitochondrial dysfunction, and restoration of klotho can alleviate these oxidative stress-related impairments. Recombinant klotho protein restored the high glucose-induced mitochondrial damage and increased OXPHOS in cultured proximal tubular cells via upregulation of renal p-AMPK and PGC1α and downregulation of mTOR/TGF-β.^64^ Likewise, ectopic expression of klotho has been shown to protect renal mitochondrial functions by preserving mitochondrial mass and inhibiting the production of ROS via Wnt/β-catenin/RAS signaling, leading to less renal fibrosis in the d-galactose-induced accelerated aging mouse model.^65^ Another study revealed that klotho markedly diminished fibrotic lesions and the activation of Wnt/β-catenin signaling along with preservation of mitochondrial mass, respiration chain complex subunits (e.g., OXPHOS complex III subunit cytochrome b), and downregulation of mitochondrial ROS.^66^ Our results were in accordance with these studies, which showed that upregulation of klotho alleviated IS-induced mitochondrial dysfunction, as evidenced by the restoration of the dysregulated membrane potential using JC-1 dye or COXIII expression as well as Complex III activity. It has been known that the inactivation of Complex III interrupts electron transport and alters the redox state by decreasing cellular NAD^+^ levels. Also, disruption of Complex III electron transport affected kidney development.^67^ We showed that the administration of klotho abrogated the effect of IS on the induction of COXIII expression and suppression of Complex III activity through AKT/Nrf2 signaling. Moreover, we demonstrated that reconstruction of the mitochondrial functional state by upregulation of klotho eliminated the IS-induced apoptosis, as the mitochondrial respiratory chain is a key modulator of apoptosis.^68^ Given that klotho exerted an anti-inflammatory effect to decrease the IS-induced kidney fibrosis^51^ and the pivotal interconnection between inflammation and apoptosis in nephropathy,^69^ whether klotho reduced apoptosis of IS-treated cells through its regulatory property in inflammation is worthy of further investigation. Klotho overexpression in animals mitigates isoproterenol-caused cardiac hypertrophic responses by inhibiting cardiac TRPC6.^70^ Our study addresses a notable gap in the literature regarding the direct effects of IS on mitochondrial function and apoptosis in renal cells and the protective role of klotho in these processes. We uniquely contribute to understanding how klotho modulates mitochondrial integrity and apoptosis via the AKT/Nrf2 pathway.

This study has several limitations. First, only pharmacological inhibitors (such as PI3K/Akt inhibitor LY294002) were used. Silencing Akt using siRNA will help us to better understand the relationship between klotho and antioxidant-related signaling pathways. Second, this study presents only the in vitro cytoprotective effects of klotho under IS exposure. The concentration of IS in our study is higher than the typical plasma concentrations observed in CKD patients. However, the higher dosage was selected to mimic the harmful effects of IS on proximal tubular cells and to explore the protective maximal impact of klotho in vitro. Although this study ensures clarity in mechanistic insights, it introduces limitations in directly translating findings into physiological conditions. Thus, we will conduct an in vivo study to comprehensively understand klotho’s protective effects and provide the translational potential.

In summary, we demonstrated that the accumulation of IS in proximal tubular cells may lead to downregulation of cell viability through the suppression of klotho. Upregulation of klotho can restore antioxidant capacity, preserve mitochondrial function, maintain the normal structure of mitochondria, and suppress apoptosis. This study has several limitations. First, our study lacks in vivo evidence, necessitating future research to validate these findings in CKD models. Second, the supplementation of klotho may be a potential avenue to abate the detrimental effects of IS in proximal renal tubule cells and ameliorate the progression of CKD.

## Abbreviations

chronic kidney disease: (CKD)
indoxyl sulfate: (IS)
nuclear factor-kappa B: (NF-κB)
fibroblast growth factor: (FGF)
nuclear factor erythroid 2-related factor 2: (Nrf2)
heme oxygenase-1: (HO-1)
NAD­(P)­H-quinone oxidoreductase 1: (NQO1)
bovine pituitary extract: (BPE)
epidermal growth factor: (EGF)
lactate dehydrogenase: (LDH)

## References

[1] Levey A. S., Atkins R., Coresh J., Cohen E. P., Collins A. J., Eckardt K. U., Nahas M. E., Jaber B. L., Jadoul M., Levin A. (2007). Chronic kidney disease as a global public health problem:
approaches and initiatives - a position statement from Kidney Disease
Improving Global Outcomes. Kidney Int..

[2] Jager K. J., Kovesdy C., Langham R., Rosenberg M., Jha V., Zoccali C. (2019). A single number for
advocacy and communication-worldwide
more than 850 million individuals have kidney diseases. Kidney Int..

[3] Sarnak M. J., Levey A. S., Schoolwerth A. C., Coresh J., Culleton B., Hamm L. L., McCullough P. A., Kasiske B. L., Kelepouris E., Klag M. J. (2003). Kidney
disease as a risk factor for development of
cardiovascular disease: a statement from the American Heart Association
Councils on Kidney in Cardiovascular Disease, High Blood Pressure
Research, Clinical Cardiology, and Epidemiology and Prevention. Circulation.

[4] GBD
2013 Mortality and Causes of Death Collaborators (2015). Global, regional, national age-sex
specific all-cause and cause-specific mortality for 240 causes of
death, 1990–2013: a systematic analysis for the Global Burden
of Disease Study 2013. Lancet.

[5] Vanholder R., Schepers E., Pletinck A., Nagler E. V., Glorieux G. (2014). The uremic
toxicity of indoxyl sulfate and p-cresyl sulfate: a systematic review. J. Am. Soc. Nephrol..

[6] Enomoto A., Takeda M., Tojo A., Sekine T., Cha S. H., Khamdang S., Takayama F., Aoyama I., Nakamura S., Endou H. (2002). Role of organic anion transporters in the tubular transport
of indoxyl sulfate and the induction of its nephrotoxicity. J. Am. Soc. Nephrol..

[7] Sirich T. L., Aronov P. A., Plummer N. S., Hostetter T. H., Meyer T. W. (2013). Numerous protein-bound solutes are
cleared by the kidney
with high efficiency. Kidney Int..

[8] Poesen R., Viaene L., Verbeke K., Claes K., Bammens B., Sprangers B., Naesens M., Vanrenterghem Y., Kuypers D., Evenepoel P. (2013). Renal clearance and
intestinal generation of p-cresyl sulfate and indoxyl sulfate in CKD. Clin. J. Am. Soc. Nephrol..

[9] Stanfel L. A., Gulyassy P. F., Jarrard E. A. (1986). Determination of indoxyl sulfate
in plasma of patients with renal failure by use of ion-pairing liquid
chromatography. Clin. Chem..

[10] Kim S. H., Yu M. A., Ryu E. S., Jang Y. H., Kang D. H. (2012). Indoxyl
sulfate-induced epithelial-to-mesenchymal transition and apoptosis
of renal tubular cells as novel mechanisms of progression of renal
disease. Lab. Invest..

[11] Motojima M., Hosokawa A., Yamato H., Muraki T., Yoshioka T. (2003). Uremic toxins
of organic anions up-regulate PAI-1 expression by induction of NF-kappaB
and free radical in proximal tubular cells. Kidney Int..

[12] Shimizu H., Bolati D., Adijiang A., Muteliefu G., Enomoto A., Nishijima F., Dateki M., Niwa T. (2011). NF-κB
plays an important role in indoxyl sulfate-induced cellular senescence,
fibrotic gene expression, and inhibition of proliferation in proximal
tubular cells. Am. J. Physiol.: Cell Physiol..

[13] Hu M. C., Shi M., Zhang J., Pastor J., Nakatani T., Lanske B., Razzaque M. S., Rosenblatt K. P., Baum M. G., Kuro-o M. (2010). Klotho:
a novel phosphaturic substance acting as an autocrine enzyme
in the renal proximal tubule. FASEB J..

[14] Kuro-o M., Matsumura Y., Aizawa H., Kawaguchi H., Suga T., Utsugi T., Ohyama Y., Kurabayashi M., Kaname T., Kume E. (1997). Mutation of the mouse
klotho gene leads to a syndrome resembling ageing. Nature.

[15] Hu M. C., Shiizaki K., Kuro-o M., Moe O. W. (2013). Fibroblast growth
factor 23 and Klotho: physiology and pathophysiology of an endocrine
network of mineral metabolism. Annu. Rev. Physiol..

[16] Kuro O. M. (2019). The Klotho
proteins in health and disease. Nat. Rev. Nephrol..

[17] Xu Y., Sun Z. (2015). Molecular basis of
Klotho: from gene to function in aging. Endocr.
Rev..

[18] Takenaka T., Kobori H., Miyazaki T., Suzuki H., Nishiyama A., Ishii N., Yamashita M., Hayashi M. (2019). Klotho protein supplementation
reduces blood pressure and renal hypertrophy in db/db mice, a model
of type 2 diabetes. Acta Physiol..

[19] Takenaka T., Inoue T., Miyazaki T., Kobori H., Nishiyama A., Ishii N., Hayashi M., Suzuki H. (2018). Klotho Ameliorates
Medullary Fibrosis and Pressure Natriuresis in Hypertensive Rat Kidneys. Hypertension.

[20] Zhu Y., Xu L., Zhang J., Xu W., Liu Y., Yin H., Lv T., An H., Liu L., He H. (2013). Klotho
suppresses tumor progression via inhibiting PI3K/Akt/GSK3β/Snail
signaling in renal cell carcinoma. Cancer Sci..

[21] Chen K., Wang S., Sun Q. W., Zhang B., Ullah M., Sun Z. (2021). Klotho Deficiency Causes
Heart Aging via Impairing the Nrf2-GR Pathway. Circ. Res..

[22] Maltese G., Psefteli P. M., Rizzo B., Srivastava S., Gnudi L., Mann G. E., Siow R. C. (2017). The anti-ageing
hormone klotho induces Nrf2-mediated antioxidant defences in human
aortic smooth muscle cells. J. Cell. Mol. Med..

[23] Wen X., Li S., Zhang Y., Zhu L., Xi X., Zhang S., Li Y. (2022). Recombinant human klotho
protects against hydrogen peroxide-mediated
injury in human retinal pigment epithelial cells via the PI3K/Akt-Nrf2/HO-1
signaling pathway. Bioengineered.

[24] Li S., Cheng S., Sun Z., Mungun H. K., Gong W., Yu J., Xia W., Zhang Y., Huang S., Zhang A. (2016). Indoxyl
Sulfate Induces Mesangial Cell Proliferation via the Induction
of COX-2. Mediators Inflammation.

[25] Cui W., Leng B., Wang G. (2019). Klotho protein inhibits H(2)­O(2)-induced
oxidative injury in endothelial cells via regulation of PI3K/AKT/Nrf2/HO-1
pathways. Can. J. Physiol. Pharmacol..

[26] Lu C. L., Zheng C. M., Lu K. C., Liao M. T., Wu K. L., Ma M. C. (2021). Indoxyl-Sulfate-Induced
Redox Imbalance in Chronic Kidney Disease. Antioxidants.

[27] Loboda A., Damulewicz M., Pyza E., Jozkowicz A., Dulak J. (2016). Role of Nrf2/HO-1 system in development, oxidative stress response
and diseases: an evolutionarily conserved mechanism. Cell. Mol. Life Sci..

[28] Granata S., Zaza G., Simone S., Villani G., Latorre D., Pontrelli P., Carella M., Schena F. P., Grandaliano G., Pertosa G. (2009). Mitochondrial dysregulation and oxidative
stress in
patients with chronic kidney disease. BMC Genomics.

[29] Capaldi R. A. (1990). Structure
and function of cytochrome c oxidase. Annu.
Rev. Biochem..

[30] Yu B. C., Cho N. J., Park S., Kim H., Choi S. J., Kim J. K., Hwang S. D., Gil H. W., Lee E. Y., Jeon J. S. (2019). IgA nephropathy is associated
with elevated urinary
mitochondrial DNA copy numbers. Sci. Rep..

[31] Eirin A., Saad A., Woollard J. R., Juncos L. A., Calhoun D. A., Tang H., Lerman A., Textor S. C., Lerman L. O. (2017). Glomerular
Hyperfiltration in Obese African American Hypertensive Patients Is
Associated With Elevated Urinary Mitochondrial-DNA Copy Number. Am. J. Hypertens..

[32] Eirin A., Saad A., Tang H., Herrmann S. M., Woollard J. R., Lerman A., Textor S. C., Lerman L. O. (2016). Urinary Mitochondrial
DNA Copy Number Identifies Chronic Renal Injury in Hypertensive Patients. Hypertension.

[33] Duranton F., Cohen G., De Smet R., Rodriguez M., Jankowski J., Vanholder R., Argiles A. (2012). Normal and pathologic
concentrations of uremic toxins. J. Am. Soc.
Nephrol..

[34] Wu I. W., Hsu K. H., Lee C. C., Sun C. Y., Hsu H. J., Tsai C. J., Tzen C. Y., Wang Y. C., Lin C. Y., Wu M. S. (2011). p-Cresyl
sulphate and indoxyl sulphate predict progression of chronic
kidney disease. Nephrol., Dial., Transplant..

[35] Nakano T., Watanabe H., Imafuku T., Tokumaru K., Fujita I., Arimura N., Maeda H., Tanaka M., Matsushita K., Fukagawa M. (2021). Indoxyl
Sulfate Contributes to mTORC1-Induced
Renal Fibrosis via The OAT/NADPH Oxidase/ROS Pathway. Toxins.

[36] Hsieh Y. H., Tsai J. P., Ting Y. H., Hung T. W., Chao W. W. (2022). Rosmarinic
acid ameliorates renal interstitial fibrosis by inhibiting the phosphorylated-AKT
mediated epithelial-mesenchymal transition in vitro and in vivo. Food Funct..

[37] Sun C. Y., Chang S. C., Wu M. S. (2012). Uremic toxins induce kidney fibrosis
by activating intrarenal renin-angiotensin-aldosterone system associated
epithelial-to-mesenchymal transition. PLoS One.

[38] Adesso S., Popolo A., Bianco G., Sorrentino R., Pinto A., Autore G., Marzocco S. (2013). The uremic
toxin indoxyl
sulphate enhances macrophage response to LPS. PLoS One.

[39] Gelasco A. K., Raymond J. R. (2006). Indoxyl sulfate
induces complex redox alterations in
mesangial cells. Am. J. Physiol. Renal Physiol..

[40] Owada S., Goto S., Bannai K., Hayashi H., Nishijima F., Niwa T. (2008). Indoxyl sulfate reduces
superoxide scavenging activity in the kidneys
of normal and uremic rats. Am. J. Nephrol..

[41] Hu M. C., Shi M., Zhang J., Quiñones H., Kuro-o M., Moe O. W. (2010). Klotho
deficiency is an early biomarker of renal ischemia-reperfusion injury
and its replacement is protective. Kidney Int..

[42] Adijiang A., Niwa T. (2010). An oral sorbent, AST-120,
increases Klotho expression and inhibits
cell senescence in the kidney of uremic rats. Am. J. Nephrol..

[43] Ide N., Olauson H., Sato T., Densmore M. J., Wang H., Hanai J. I., Larsson T. E., Lanske B. (2016). In vivo evidence for
a limited role of proximal tubular Klotho in renal phosphate handling. Kidney Int..

[44] Hu M. C., Shi M., Zhang J., Quiñones H., Griffith C., Kuro-o M., Moe O. W. (2011). Klotho deficiency
causes vascular calcification in
chronic kidney disease. J. Am. Soc. Nephrol..

[45] Sugiura H., Yoshida T., Shiohira S., Kohei J., Mitobe M., Kurosu H., Kuro-o M., Nitta K., Tsuchiya K. (2012). Reduced Klotho
expression level in kidney aggravates renal interstitial fibrosis. Am. J. Physiol. Renal Physiol..

[46] Zhao Y., Banerjee S., Dey N., LeJeune W. S., Sarkar P. S., Brobey R., Rosenblatt K. P., Tilton R. G., Choudhary S. (2011). Klotho depletion
contributes to increased inflammation in kidney of the db/db mouse
model of diabetes via RelA (serine)­536 phosphorylation. Diabetes.

[47] Shimizu H., Bolati D., Adijiang A., Adelibieke Y., Muteliefu G., Enomoto A., Higashiyama Y., Higuchi Y., Nishijima F., Niwa T. (2011). Indoxyl sulfate downregulates
renal expression of Klotho through production of ROS and activation
of nuclear factor-KB. Am. J. Nephrol..

[48] Sun C. Y., Chang S. C., Wu M. S. (2012). Suppression
of Klotho expression
by protein-bound uremic toxins is associated with increased DNA methyltransferase
expression and DNA hypermethylation. Kidney
Int..

[49] Koh N., Fujimori T., Nishiguchi S., Tamori A., Shiomi S., Nakatani T., Sugimura K., Kishimoto T., Kinoshita S., Kuroki T. (2001). Severely
reduced production
of klotho in human chronic renal failure kidney. Biochem. Biophys. Res. Commun..

[50] Adijiang A., Shimizu H., Higuchi Y., Nishijima F., Niwa T. (2011). Indoxyl sulfate reduces klotho expression and promotes senescence
in the kidneys of hypertensive rats. J. Renal
Nutr..

[51] Lv J., Chen J., Wang M., Yan F. (2020). Klotho alleviates indoxyl
sulfate-induced heart failure and kidney damage by promoting M2 macrophage
polarization. Aging.

[52] Yang K., Nie L., Huang Y., Zhang J., Xiao T., Guan X., Zhao J. (2012). Amelioration of uremic
toxin indoxyl sulfate-induced endothelial
cell dysfunction by Klotho protein. Toxicol.
Lett..

[53] Yang K., Wang C., Nie L., Zhao X., Gu J., Guan X., Wang S., Xiao T., Xu X., He T. (2015). Klotho
Protects Against Indoxyl Sulphate-Induced Myocardial
Hypertrophy. J. Am. Soc. Nephrol..

[54] Hu M. C., Shi M., Gillings N., Flores B., Takahashi M., Kuro O. M., Moe O. W. (2017). Recombinant
alpha-Klotho may be prophylactic
and therapeutic for acute to chronic kidney disease progression and
uremic cardiomyopathy. Kidney Int..

[55] Xie J., Yoon J., An S. W., Kuro-o M., Huang C. L. (2015). Soluble
Klotho Protects against Uremic Cardiomyopathy Independently of Fibroblast
Growth Factor 23 and Phosphate. J. Am. Soc.
Nephrol..

[56] He T., Xiong J., Huang Y., Zheng C., Liu Y., Bi X., Liu C., Han W., Yang K., Xiao T. (2019). Klotho
restrain RIG-1/NF-κB signaling activation and monocyte
inflammatory factor release under uremic condition. Life Sci..

[57] Pan H. C., Chou K. M., Lee C. C., Yang N. I., Sun C. Y. (2018). Circulating
Klotho levels can predict long-term macrovascular outcomes in type
2 diabetic patients. Atherosclerosis.

[58] Yang K., Du C., Wang X., Li F., Xu Y., Wang S., Chen S., Chen F., Shen M., Chen M. (2017). Indoxyl sulfate induces
platelet hyperactivity and contributes to
chronic kidney disease-associated thrombosis in mice. Blood.

[59] Jin J., Jin L., Lim S. W., Yang C. W. (2016). Klotho Deficiency
Aggravates Tacrolimus-Induced
Renal Injury via the Phosphatidylinositol 3-Kinase-Akt-Forkhead Box
Protein O Pathway. Am. J. Nephrol..

[60] Lim S. W., Shin Y. J., Luo K., Quan Y., Cui S., Ko E. J., Chung B. H., Yang C. W. (2019). Ginseng increases
Klotho expression by FoxO3-mediated manganese superoxide dismutase
in a mouse model of tacrolimus-induced renal injury. Aging.

[61] Zhao M., Murakami S., Matsumaru D., Kawauchi T., Nabeshima Y. I., Motohashi H. (2022). NRF2 pathway activation attenuates ageing-related renal
phenotypes due to α-klotho deficiency. J. Biochem..

[62] Xing L., Guo H., Meng S., Zhu B., Fang J., Huang J., Chen J., Wang Y., Wang L., Yao X. (2021). Klotho ameliorates diabetic
nephropathy by activating Nrf2 signaling
pathway in podocytes. Biochem. Biophys. Res.
Commun..

[63] Tirichen H., Yaigoub H., Xu W., Wu C., Li R., Li Y. (2021). Mitochondrial Reactive Oxygen Species
and Their Contribution in Chronic
Kidney Disease Progression Through Oxidative Stress. Front. Physiol..

[64] Lee J., Tsogbadrakh B., Yang S., Ryu H., Kang E., Kang M., Kang H. G., Ahn C., Oh K. H. (2021). Klotho
ameliorates diabetic nephropathy via LKB1-AMPK-PGC1α-mediated
renal mitochondrial protection. Biochem. Biophys.
Res. Commun..

[65] Miao J., Liu J., Niu J., Zhang Y., Shen W., Luo C., Liu Y., Li C., Li H., Yang P. (2019). Wnt/β-catenin/RAS
signaling mediates age-related renal fibrosis and is associated with
mitochondrial dysfunction. Aging Cell.

[66] Miao J., Huang J., Luo C., Ye H., Ling X., Wu Q., Shen W., Zhou L. (2021). Klotho retards
renal fibrosis through
targeting mitochondrial dysfunction and cellular senescence in renal
tubular cells. Physiol. Rep..

[67] Guan N., Kobayashi H., Ishii K., Davidoff O., Sha F., Ikizler T. A., Hao C. M., Chandel N. S., Haase V. H. (2022). Disruption
of mitochondrial complex III in cap mesenchyme but not in ureteric
progenitors results in defective nephrogenesis associated with amino
acid deficiency. Kidney Int..

[68] Kwong J. Q., Henning M. S., Starkov A. A., Manfredi G. (2007). The mitochondrial respiratory
chain is a modulator of apoptosis. J. Cell Biol..

[69] Sanz A. B., Sanchez-Niño M. D., Ramos A. M., Ortiz A. (2023). Regulated
cell death pathways in kidney disease. Nat.
Rev. Nephrol..

[70] Xie J., Cha S. K., An S. W., Kuro-o M., Birnbaumer L., Huang C. L. (2012). Cardioprotection
by Klotho through downregulation of
TRPC6 channels in the mouse heart. Nat. Commun..

